# Mitigative Effects of PFF-A Isolated from *Ecklonia cava* on Pigmentation in a Zebrafish Model and Melanogenesis in B16F10 Cells

**DOI:** 10.3390/md20020123

**Published:** 2022-02-04

**Authors:** Jun-Geon Je, Yunfei Jiang, Jun-Ho Heo, Xining Li, You-Jin Jeon, Bo-Mi Ryu

**Affiliations:** 1 Department of Marine Life Sciences, Jeju National University, Jeju 63243, Korea; wpwnsrjs3@jejunu.ac.kr (J.-G.J.); unknown0713@stu.jejunu.ac.kr (J.-H.H.); youjinj@jejunu.ac.kr (Y.-J.J.); 2 School of Food Engineering, Jilin Agriculture Science and Technology University, Jilin 132101, China; jiangyunfei@jlnku.edu.cn; 3 School of Life Sciences, Northeast Normal University, Changchun 130024, China; lixn887@nenu.edu.cn; 4 Marine Science Institute, Jeju National University, Jeju 63333, Korea

**Keywords:** phlorofucofuroeckol-A, *Ecklonia cava*, melanogenesis, B16F10 cell lines, zebrafish

## Abstract

Melanin synthesis is a defense mechanism that prevents skin damage, but excessive accumulation of melanin occurs in the skin in various reactions such as pigmentation, lentigines, and freckles. Although anti-melanogenic effects have been demonstrated for various naturally occurring marine products that inhibit and control tyrosinase activity, most studies have not been extended to in vivo applications. Phlorofucofuroeckol-A (PFF-A, 12.5–100 µM) isolated from *Ecklonia cava* has previously been shown to have tyrosinase-mitigative effects in B16F10 cells, but it has not been evaluated in an in vivo model, and its underlying mechanism for anti-melanogenic effects has not been studied. In the present study, we evaluated the safety and efficacy of PFF-A for anti-melanogenic effects in an in vivo model. We selected low doses of PFF-A (1.5–15 nM) and investigated their mitigative effects on pigmentation stimulated by α-MSH in vivo and their related-mechanism in an in vitro model. The findings suggest that low-dose PFF-A derived from *E. cava* suppresses pigmentation in vivo and melanogenesis in vitro. Therefore, this study presents the possibility that PFF-A could be utilized as a new anti-melanogenic agent in the cosmeceutical industries.

## 1. Introduction

Melanin synthesis, also known as melanogenesis, is a defense mechanism that prevents ultraviolet (UV)-radiation-induced skin damage. Normal melanin pigmentation is produced by melanocytes to inhibit dermal degradation and to protect skin cells from UV-radiation-induced damage [1,2,3]. However, excess UV exposure increases the accumulation of melanin, which causes skin pigmentation and visible skin pigmentation phenotypes such as lentigines and freckles [4,5].

There have been many previous studies using animal models of melanogenesis [6,7,8]. Zebrafish are models used globally in toxicity and biomedical studies, and their embryos are particularly useful as they can serve as an alternative to other animal models [9]. The zebrafish model has been suggested as an alternative to mammalian models, due to several advantages, including rapid model development, cost efficiency, and physiological relevance. In addition, the zebrafish has emerged as a useful vertebrate model organism because of its physiological similarity with mammals, and as there is melanin pigment on its surface, pigmentation processes can be easily observed even if there is no complicated experiment [10,11]. Therefore, the zebrafish model can generate useful data for adaptation in both in vivo models and clinical studies, and can be used to generate data to inform future research [12,13].

Tyrosinase is a copper-ion enzyme that is present in a wide variety of microorganisms, plants, and animals. It is a crucial enzyme in regulating the metabolic pathway of melanin formation [14]. Tyrosinase catalyzes melanin synthesis in two distinct reactions in which L-tyrosine is first hydroxylated to l-DOPA via monophenolase activity, and then l-DOPA is further oxidized into dopaquinone [2,15]. MITF, a key regulator of melanogenesis signaling, inhibits the expression of tyrosinase and TRP-1, which results in lower levels of melanin production and may be regarded as a strategy to inhibit melanogenesis [16]. Recently, several studies have focused on the effect of protein kinase B (Akt) on the melanogenesis pathway and demonstrated the acceleration of Akt-mediated MITF degradation [17,18]. Natural products that trigger signaling pathways involved in melanin biosynthesis and transformation may be useful in the development of anti-melanogenic agents for both medical and cosmetic applications.

Previous studies have reported that marine brown algae have anti-melanogenic effects through tyrosinase inhibition [19]. *Ecklonia cava* has been reported to inhibit tyrosinase and other TRPs related to melanin production, and to have potential for use in melanin-related drugs or cosmetics in the future [15]. PFF-A, a phlorotannin isolated from *E. cava*, has been shown to exhibit anti-tyrosinase activity and has a unique structure and various physiological activities [19,20,21]. Phlorotannins are hydrophilic compounds with a wide range of molecular sizes between 162 Da and 650 kDa, and they have strong structure-dependent tyrosinase inhibitory activity in the order of trimer > dimer > monomer [22]. Previous studies have shown potential effects of PFF-A up to 100 μM in α-MSH-treated B16F10 cells [23]. However, these properties of PFF-A do not translate to in vivo models because of the adverse effects of overdose, and thus the related mechanisms remain elusive.

In this study, low doses of PFF-A derived from *E. cava* were investigated for their anti-melanogenic effects, with a focus on tyrosinase inhibition activity and depigmentation in vivo and in vitro. Furthermore, we measured the amount of accumulated melanin after α-MSH stimulation in zebrafish skin and verified the anti-melanogenic effect and the mechanism of action of PFF-A in B16F10 cells. Therefore, we demonstrated the anti-melanogenic effect of PFF-A in zebrafish and expanded its potential application as an anti-melanogenic agent in the cosmetic and pharmaceutical industries.

## 2. Results and Discussion

### 2.1. Melanin Synthesis Mitigative Activity of Low Doses of PFF-A in Zebrafish Larvae

Zebrafish is useful as a vertebrate model organism because it has a human-like organ system and genome sequence [24]. In this study, zebrafish were used as an in vivo animal model to examine the depigmentation property of tyrosinase inhibitors [25]. Using this model, melanin pigments accumulated on the surface of zebrafish, allowing the process of melanin synthesis to be observed under a microscope without complicated experimental procedures [26]. The zebrafish model has been used in an expanded in vitro study and is widely used for in vivo models in clinical studies [27,28,29]. The anti-tyrosinase activity of PFF-A (12.5–100 µM) isolated from *Ecklonia stolonifera* has previously been reported in a preclinical study [23]. Although the possibility of using PFF-A as a tyrosinase inhibitor was suggested, the study was limited to in vitro experiments because of the lack of studies in zebrafish and other in vivo models, and thus the applicability of this finding to humans is low. Therefore, in this study we extended our in vivo studies using a zebrafish model to confirm the anti-melanogenesis effect of PFF-A. We investigated the anti-melanogenic activity of low doses of PFF-A isolated from *E. cava* in an in vivo zebrafish model.

**Figure 1:**
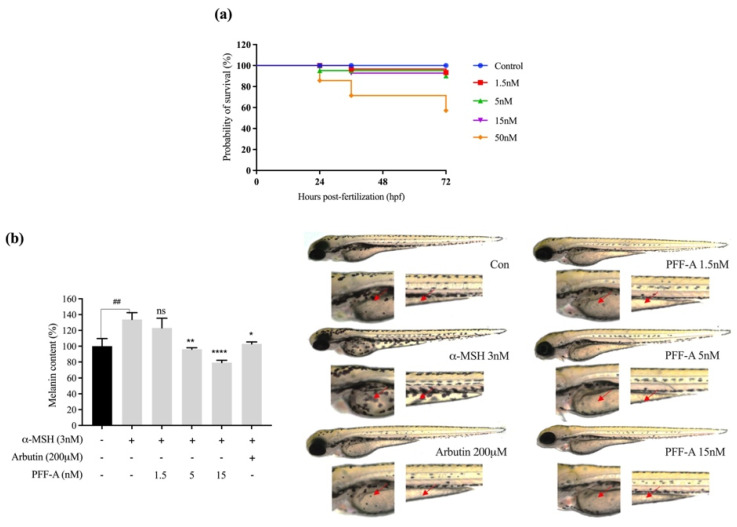
Effects of phlorofucofuroeckol-A (PFF-A) on pigment accumulation in zebrafish. Synchronized larvae were treated with PFF-A for 72 h. The positive control was arbutin for measuring melanin content. (**a**) Survival rate after PFF-A treatment of the in vivo zebrafish model. (**b**) Melanin content in zebrafish after treatment with different concentrations of PFF-A. Images of the zebrafish larva’s body captured using a microscope (Lionheart FX, BioTek, 4×). Data are normalized to the α-MSH group and are presented as the mean ± standard deviation (SD), *n* = 3. ^##^ *p* < 0.01 vs. α-MSH-nontreated group; * *p* < 0.05, ** *p* < 0.01, and **** *p* < 0.0001 vs. α-MSH-treated group; ns, non-significant.

The toxicity of PFF-A is shown in Figure 1. As shown in Figure 1a, treatment with 50 nM PFF-A resulted in a lower survival rate; therefore, the 50 nM PFF-A dose was not included in subsequent experiments. Melanin synthesis was assessed in zebrafish larvae. The toxicity of PFF-A was examined at different concentrations (1.5, 5, 15, and 50 nM) in zebrafish larvae. Toxicity was detected at 50 nM PFF-A, whereas no change in the survival rate of zebrafish was seen at 1.5, 5, or 15 nM PFF-A. Therefore, 1.5–15 nM PFF-A was selected as the treatment range for subsequent melanogenesis studies in zebrafish larvae. The effect of PFF-A on zebrafish pigmentation was also examined. As a positive control, arbutin, a tyrosinase inhibitor commonly used to suppress pigment production in zebrafish, was also included [10,30]. At a concentration of 5 nM, PFF-A treatment inhibited melanogenesis more than arbutin treatment (102.8% of normal melanin content). As shown in Figure 1b, upon treatment with α-MSH, visible melanocytes clustered at the yolk and ventral stripes, whereas PFF-A considerably decreased the accumulation of melanin in α-MSH-treated zebrafish embryos (red arrows). According to results, melanin content increased to 130.4% of normal levels after treatment with α-MSH, and melanin accumulation was significantly decreased to 96.2% and 79.2% following 5 and 15 nM PFF-A treatment, respectively, whereas 1.5 nM of PFF-A did not lead to significant decreases.

As 15 nM PFF-A showed toxicity in the zebrafish model, non-toxic concentrations of PFF-A were applied and the reduction in melanin content in the skin was investigated. These results indicate that low doses of PFF-A significantly inhibited melanin formation in the skin of zebrafish, which reduced the number of punctate melanocytes in the zebrafish model.

### 2.2. Effects of Low Doses of PFF-A on Melanin Synthesis in B16F10 Cells and Tyrosinase Activity and Melanin Synthesis in α-MSH-treated B16F10 Cells

Based on the toxicity of PFF-A in the zebrafish model, we investigated the effect of low doses of PFF-A (1.5, 5, and 15 nM) on B16F10 cells stimulated with α-MSH.

**Figure 2:**
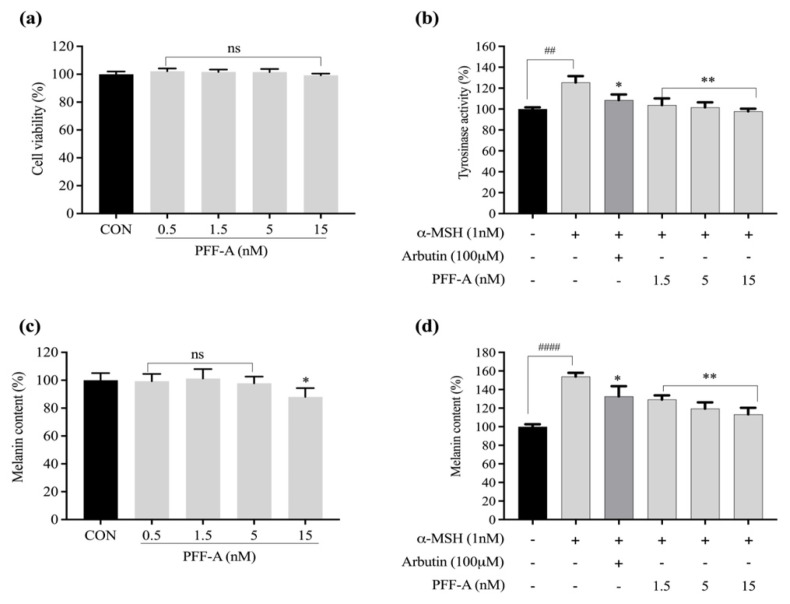
Mitigative effects of PFF-A on tyrosinase and melanin biosynthesis. (**a**) B16F10 cell viability after treatment with various concentrations of PFF-A. (**b**) Tyrosinase-mitigative activity of PFF-A. (**c**) Melanin content in B16F10 cells after treatment with various concentrations of PFF-A. (**d**) The effect of PFF-A on melanin synthesis in α-MSH-treated B16F10 cells. Data are normalized to the α-MSH group and are presented as the mean ± SD, *n* = 3. ^##^ *p* < 0.01, ^####^ *p* < 0.0001 vs. α-MSH-nontreated group; * *p* < 0.05, ** *p* < 0.01 vs. α-MSH-treated group; ns, non-significant.

To determine the cytotoxic effects of PFF-A-treated B16F10 cells, cell viability was assessed by the MTT assay. As shown in Figure 2a, there was no cytotoxicity at the tested concentrations of PFF-A (0.5, 1.5, 5, and 15 nM). The tyrosinase-mitigative effects of PFF-A were evaluated in α-MSH-treated B16F10 cells. As shown in Figure 2b, tyrosinase activity increased to 125.3% of control (without α-MSH treatment) levels after treatment with 1 nM α-MSH. Tyrosinase activity was reduced to 103.8%, 101.7%, and 97.8% of control levels after treatment of MSH-treated B16F10 cells with 1.5, 5, and 15 nM of PFF-A, respectively. Arbutin treatment reduced tyrosinase activity to 108.5% of control levels in these cells.

The melanin content of B16F10 cells was 88.1% of control levels after treatment with 15 nM PFF-A (Figure 2c). Thus, 1.5, 5, and 15 nM PFF-A were used in the melanin synthesis assay. The results presented in Figure 2d show that the melanin content increased to 153.9% of control levels after α-MSH treatment, and this was reduced to 129.2%, 119.5%, and 113.3% after treatment with 1.5, 5, and 15 nM PFF-A, respectively. Based on these results, PFF-A significantly inhibited melanin synthesis and tyrosinase activity.

### 2.3. Low Doses of PFF-A Inhibit Melanogenesis by Regulating MITF Levels and Tyrosinase Activity via the PI3K/Akt Signaling in α-MSH-Exposed B16F10 Cells

**Figure 3:**
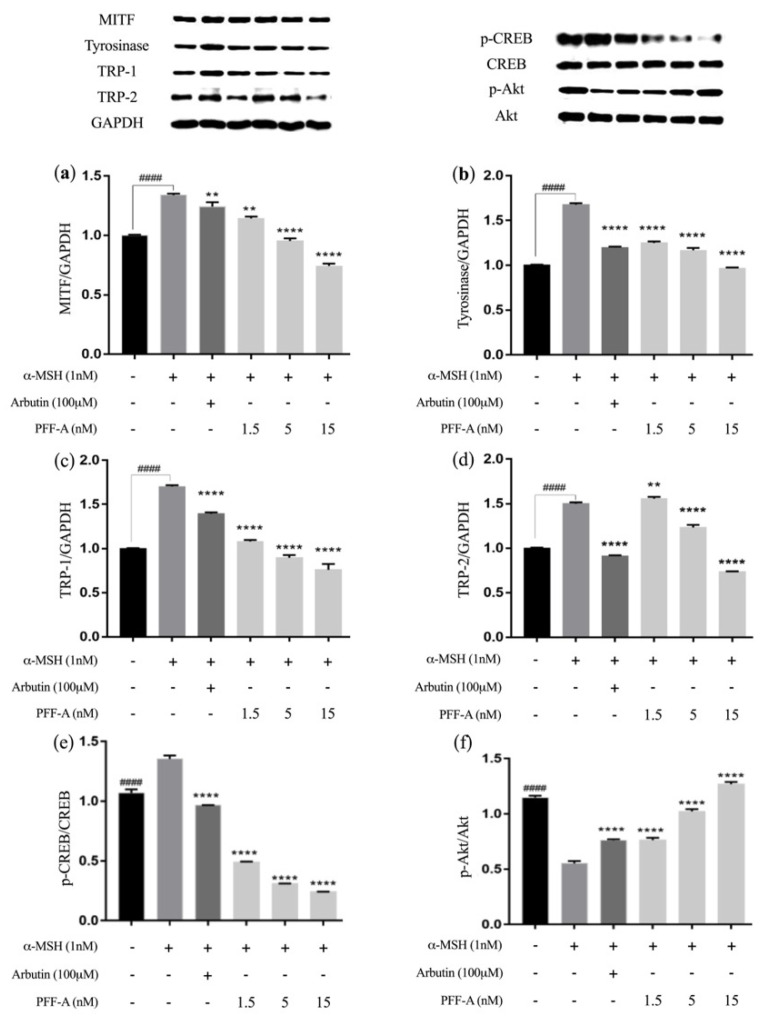
Effects of PFF-A on melanogenic proteins and signaling pathways in B16F10 cells. The expression of (**a**) MITF, (**b**) tyrosinase, (**c**) TRP-1, (**d**) TRP-2, (**e**) p-CREB/CREB, and (**f**) p-Akt/Akt proteins was evaluated by Western blotting. The Western blot bands are normalized by control group (set to 1) and are presented as the mean ± SD, *n* = 3. ^####^ *p* < 0.0001 vs. α-MSH-nontreated group; ** *p* < 0.01, and **** *p* < 0.0001 vs. α-MSH-treated group.

In earlier studies, many proteins have been reported on regarding their whitening effects resulting from the suppression of tyrosinase and MITF, which is a master regulator of melanogenesis and is involved in the induction of melanoma and melanocyte differentiation, as well as the expression of the melanogenesis-related proteins TRP-1 and TRP-2 [31,32]. To determine whether lower concentrations of PFF-A (1.5, 5, and 15 nM) also regulate the expression of these proteins, we performed Western blotting assays to evaluate the effects of PFF-A on the expression of melanogenesis-associated proteins. As can be seen in Figure 3a–d, the expressions of MITF, tyrosinase, TRP-1, and TRP-2 substantially increased after α-MSH induction. Accordingly, it was also found that PFF-A treatment significantly reduced the levels of the melanogenesis-specific proteins MITF, tyrosinase, TRP-1, and TRP-2 in B16F10 cells in a dose-dependent manner.

We further examined whether MITF was regulated by PFF-A via CREB/Akt signaling (Figure 3e,f). Treatment with PFF-A reduced the phosphorylation levels of CREB that were stimulated by α-MSH. Additionally, the levels of p-Akt were downregulated by α-MSH treatment but upregulated by PFF-A treatment. The Akt pathway and the production of intracellular cAMP regulate MITF expression by inhibiting PI3K [33]. Moreover, Vachtenheim et al. (2010) reported that melanogenic protein expressions (tyrosinase, TRP-1 and TRP-2) were highly regulated by a MITF-mediated melanogenic promoter [34].

During melanogenesis, PFF-A significantly attenuated MITF expression levels by upregulating the phosphorylation of members of the Akt pathway. Moreover, the expression level of tyrosinase was reduced by PFF-A treatment. These results indicate that PFF-A suppressed melanin synthesis by down-regulating the expression of melanogenic proteins in B16F10 cells.

## 3. Materials and Methods

### 3.1. Chemicals and Reagents

DMSO, MTT, L-DOPA, α-MSH, and arbutin were purchased from Sigma–Aldrich (St. Louis, MO, USA). Primary antibodies, including anti-p-Akt (sc-514032) and anti-tyrosinase (sc-73244) antibodies, were obtained from Santa Cruz Biotechnology (Dallas, TX, USA). MITF (D5G7V) was purchased from Cell Signaling Technology (Danvers, MA, USA). Secondary antibodies (anti-rabbit IgG and anti-mouse IgG) were purchased from Thermo Fisher Scientific (Waltham, MA, USA). All reagents were of analytical grade.

### 3.2. Preparation and Isolation of PFF-A

*E. cava* powder (100 g) was soaked in 3 L of 50% ethanol and stirred at 190× *g* for 24 h at room temperature. The solvent was then decanted, filtered, and concentrated using a rotary evaporator to obtain an ethanol extract (35.9 g). The ethanolic crude extract was extracted with n-hexane, dichloromethane, and ethyl acetate separately to obtain n-hexane-soluble (1.9 g), dichloromethane (6.2 g), and ethyl acetate (5.8 g) fractions, respectively. The ethyl acetate fraction (0.5 g) was isolated using a pure chromatography system (C-850 Flash Prep; Buchi, Flawil, Switzerland). The mobile phase system was MeOH and distilled water, and the ethyl acetate fraction was loaded onto a FlashPure cartridge (FP ECOFLEX C18, 40 g; Buchi). A 30% MeOH fraction was obtained and evaporated until dry. The 30% MeOH fraction (25 mg) was then loaded onto an HPLC column (YMC-Pack ODS-A 250 × 20.0 mml.D. S-5 µm, 12 nm AA12S05-2520WT, YMC Co., Ltd., Kyoto, Japan) and analyzed on a pure chromatography system.

The *E. cava* extract was analyzed by reverse-phase HPLC to determine the PFF-A content. HPLC analysis was performed on a Waters HPLC system with a mobile phase consisting of acetonitrile–water and an isocratic method (30 min, 30:70 *v*/*v*). The column was a Poroshell 120 EC-C18 column (4 μm 4.6 × 100 mm; Agilent, Santa Clara, CA, USA), and the flow rate was 0.5 mL/min. The results were recorded in the 230 nm range. As shown in Supplementary Figure S1, the PFF-A content was 41.3 ± 1.0 mg/g.

### 3.3. Determination of Tyrosinase Activity

Tyrosinase inhibitory activity was evaluated using a previously described method with slight modifications [35]. Briefly, a reaction mixture was prepared containing 1.5 mM L-tyrosine (40 μL), 50 mM phosphate buffer (140 μL, pH 6.5), and 10 μL of the sample solution. A tyrosinase solution (10 μL, 1500 units/mL) was then added to each well and samples were incubated at 37 °C for 12 min. The reaction was monitored at 495 nm using a BioTek Synergy HT microplate reader (BioTek Instruments, Winooski, VT, USA).

### 3.4. In Vivo Assays

#### 3.4.1. Origin and Maintenance of Parental Zebrafish

Adult zebrafish were purchased from a wholesaler (Seoul Aquarium, Seoul, Korea) and maintained at 28 °C in a temperature-controlled room with a 14/10 h day/night cycle. The day before the experiment was performed, zebrafish were randomly separated for mating in a male-to-female ratio of 2:1. The embryos were collected apart from the breeding box at the bottom of the tank and washed to remove any debris. The embryos obtained post-spawning were staged and dispensed into an embryo medium, which contained 60 mg/L Instant Ocean (Blacksburg, VA, USA) in deionized water [36,37]. The animal experiment including zebrafish models was permitted by the Animal Care and Use Committee of Jeju National University (Approval No. 2020-0049).

#### 3.4.2. Zebrafish Pigmentation Measurement

The depigmentation effect was determined following a previously published method [2,38], with slight modifications. Briefly, 12-well plates with 15 zebrafish embryos per well were seeded with 1.9 mL of embryo culture media. The PFF-A was dissolved in 1% DMSO containing 1× PBS and was used to treat the zebrafish embryos. The surviving embryos were counted every morning for 5 days to determine the survival rate. To measure melanin synthesis, after 72 h of incubation, 30 embryos were collected in 1.5 mL Eppendorf tubes and melanin production in zebrafish skin was measured under a microscope (Lionheart FX, BioTek). After acquiring images, the zebrafish were killed by placing them at −80 °C for 10 min. The embryos were then lysed in lysis buffer (1% Triton X-100 with 50 mM PBS and 1 mM PMSF) for 20 min and then clarified by centrifuge at 15,540× *g* for 30 min at 4 °C. The protein content of the lysate was determined using the Bradford assay, with bovine serum albumin as the standard. The cell lysate was dissolved in 400 µL of 1 M NaOH at 95 °C for 30 min, and the melanin content was detected by a microplate reader (BioTek) at 450 nm absorbance.

### 3.5. Cell Assay

The B16F10 mouse melanoma cell line was obtained from the American Type Culture Collection (Washington, DC, USA). Cells were cultured in Dulbecco’s modified Eagle’s medium (Gibco, Carlsbad, CA, USA) supplemented with 10% fetal bovine serum and 1% penicillin-streptomycin at 37 °C in a humidified incubator with 5% CO_2_. B16F10 cells were passaged under 10.

#### 3.5.1. Determination of Melanin Content

Before measuring the effect of PFF-A on tyrosinase activity and melanin content, its toxicity to B16F10 cells was measured. B16F10 cells were seeded (2 × 10^4^ cells/mL) in 96-well plates and, after 24 h, the cells were treated with various concentrations of PFF-A and cell viability was determined using the MTT assay, following a previously described method [15,39]. Melanin content was determined by a modified method from Hosoi et al. (1985) [40]. In brief, B16F10 cells were plated in 24-well plates at 2 × 10^4^ cells/mL and treated with PFF-A in the presence or absence of α-MSH for 48 h. Cell pellets were harvested after washing with PBS, liquefied in 1 N NaOH at 60 °C for 1 h, and mixed. Melanin content was determined by measuring the absorbance at 450 nm using a microplate reader (BioTek). For the accurate calculation of melanin content, absorbance values were normalized to total protein absorbance values. Arbutin (200 μM) was selected as a positive control.

#### 3.5.2. Cellular Tyrosinase Mitigative Activity

The inhibitory effect on tyrosinase was measured as previously described [41]. Briefly, B16F10 cells were treated with PFF-A in the presence or absence of α-MSH and, after 48 h of incubation, the cells were washed with cold PBS, suspended in lysis buffer (20 mM Tris, 5 mM EDTA, 10 mM Na_4_P_2_O_7_, 100 mM NaF, 2 mM Na_3_VO_4_, 1% NP-40, 10 mg/mL aprotinin) containing 1.0% Triton-X 100 and protease inhibitors (1 μg/mL leupeptin and 1 mM PMSF), and incubated at 4 °C for 20 min to obtain cell lysates. The cell lysates were then centrifuged at 18,600× *g* for 10 min at 4 °C. The protein content in the supernatants was then determined using a bicinchoninic acid protein assay kit. Then, cell extract was transferred to a 96-well plate containing L-DOPA (final concentration of 1 mmol/L) prepared in 25 mM phosphate buffer (pH 6.8) and incubated at 37 °C for 20 min. The optimal absorbance was at 475 nm (BioTek).

#### 3.5.3. Western Blotting Analysis

B16F10 melanoma cells were plated in culture dishes with or without PFF-A for 48 h and then lysed in lysis buffer. Equal amounts of protein were electrophoresed through 10% sodium dodecyl sulfate-poly acrylamide gels. The separated proteins were then transferred onto PVDF membranes. The membranes were then incubated with primary antibodies (anti-p-Akt/Akt, anti-MITF, and anti-tyrosinase; 1:1000 dilution) at 4 °C for 8 h. The blots were washed multiple times with Tween 20/Tris-buffered saline and incubated with their secondary antibodies for 45 min (1:3000) at room temperature. The protein bands on the PVDF membranes were detected using an enhanced chemiluminescent substrate. Membrane images were captured using a FUSION SOLO system (Vilber Lourmat, Collegien, France). Signal intensities of the protein bands were determined by densitometry using ImageJ software (version 1.4; National Institutes of Health, Bethesda, MD, USA).

### 3.6. Statistical Analysis

Data were analyzed by two-way analysis of variance and Dunnett’s multiple range tests using GraphPad Prism 9.0 software (GraphPad, San Diego, CA, USA). All experiments were performed three times and the results are expressed as the mean ± standard deviation.

## 4. Conclusions

In this study, the toxicity and efficacy of PFF-A isolated from *E. cava* were evaluated in a zebrafish model. Considering safety and efficacy, our study showed that low doses of PFF-A (1.5–15 nM) inhibited the overwhelmed pigmentation in zebrafish skin. We then examined the effect of low doses of PFF-A on melanin formation and suggested the role of PFF-A in the regulation of MITF/CREB signaling-related proteins in B16F10 cells. The anti-melanogenic effect of PFF-A isolated from *E. cava* observed in this study suggests that clinical studies assessing PFF-A are warranted and thereafter, PFF-A may be considered for anti-melanin applications in the pharmaceutical and cosmetic industries.

## Supplementary Materials

The following supporting information can be downloaded at: https://www.mdpi.com/article/10.3390/md20020123/s1, Figure S1: HPLC chromatogram. (a) Phlorofucofuroeckol-A (PFF-A, 1 mg/mL) (b) *Ecklonia cava* ethanol extract (ECE, 5 mg/mL).

## Abbreviations

α-Melanocyte-stimulating hormone (α-MSH); 3,4-dihydroxyphenylalanine (L-DOPA); microphthalmia-associated transcription factor (MITF); tyrosinase-related protein-1 (TRP-1); 3-(4,5-dimethylthiazol-2-yl)-2,5-diphenyltetrazolium bromide (MTT); cyclic adenosine monophosphate (cAMP); cyclic adenosine monophosphate-response element binding protein (CREB); phosphatidylinositol 3-kinase (PI3K); dimethyl sulfoxide (DMSO); high-performance liquid chromatography (HPLC); phosphate-buffered saline (PBS); phenyl methyl sulfonyl fluoride (PMSF); polyvinylidene fluoride (PVDF).
