# Supplementary material for: Mitigative Effects of PFF-A Isolated from Ecklonia cava on Pigmentation in a Zebrafish Model and Melanogenesis in B16F10 Cells
Source: Mar Drugs. 2022 Feb 4;20(2):123. doi: 10.3390/md20020123 (PMC8877154; doi:10.3390/md20020123)
Supplement: Supplementary file 1 [file marinedrugs-20-00123-s001.zip › marinedrugs-1585100-supplementary.pdf]

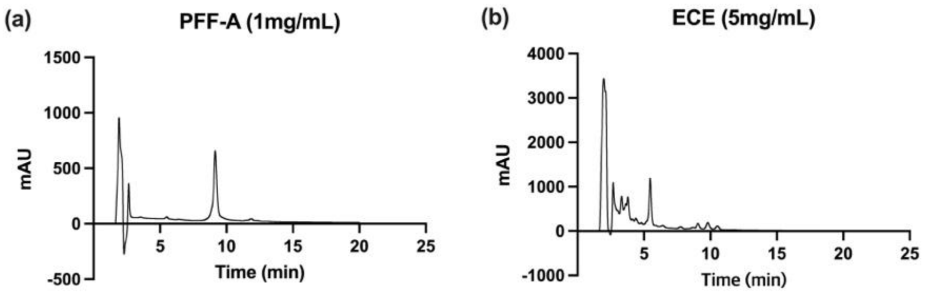

Supplementary Figure S1. HPLC chromatogram. (a) Phlorofucofuroeckol-A (PFF-A, 1mg/mL) (b) *Ecklonia cava* 50% ethanol extract (ECE, 5mg/mL)
